# Supplementary material for: Cytosolic N6AMT1-dependent translation supports mitochondrial RNA processing
Source: Proc Natl Acad Sci U S A. 2024 Nov 6;121(47):e2414187121. doi: 10.1073/pnas.2414187121 (PMC11588129; doi:10.1073/pnas.2414187121)
Supplement: Supplementary file 1 — Appendix 01 (PDF) [file pnas.2414187121.sapp.pdf]

PNAS

Supporting Information for

Cytosolic N6AMT1-dependent translation supports mitochondrial RNA processing

Mads M. Foged[a], Emeline Recazens[a,1], Sylvain Chollet[a,1], Miriam Lisci[a], George E. Allen[b], Boris Zinshteyn[c,d], Doha Boutguetait[e], Christian Münch[e], Vamsi K. Mootha[f,g,h,i] and Alexis A. Jourdain[a,2]

[a]Department of Immunobiology, University of Lausanne, 1066 Epalinges, Switzerland

[b]Department of Microbiology and Molecular Medicine, Institute of Genetics and Genomics Geneva, Faculty of Medicine, University of Geneva, 1211 Geneva 4, Switzerland

[c]Howard Hughes Medical Institute, Johns Hopkins University School of Medicine, Baltimore, MD 21205

[d]Department of Molecular Biology and Genetics, Johns Hopkins University School of Medicine, Baltimore, MD 21205

[e]Institute of Molecular Systems Medicine, Faculty of Medicine, Goethe University Frankfurt, 60590 Frankfurt am Main, Germany

[f]Broad Institute of Massachusetts Institute of Technology and Harvard, Cambridge, MA 02142

[g]Howard Hughes Medical Institute, Massachusetts General Hospital Boston, MA 02114

[h]Department of Molecular Biology, Massachusetts General Hospital, Boston, MA 02114

[i]Department of Systems Biology, Harvard Medical School, Boston, MA 02115

[1]Co-second authors

[2]Corresponding author: Alexis A. Jourdain

Email: alexis.jourdain@unil.ch

This PDF file includes:

Figures S1 to S5
Tables S1 to S4

Other supporting materials for this manuscript include the following:

Datasets S1 to S13

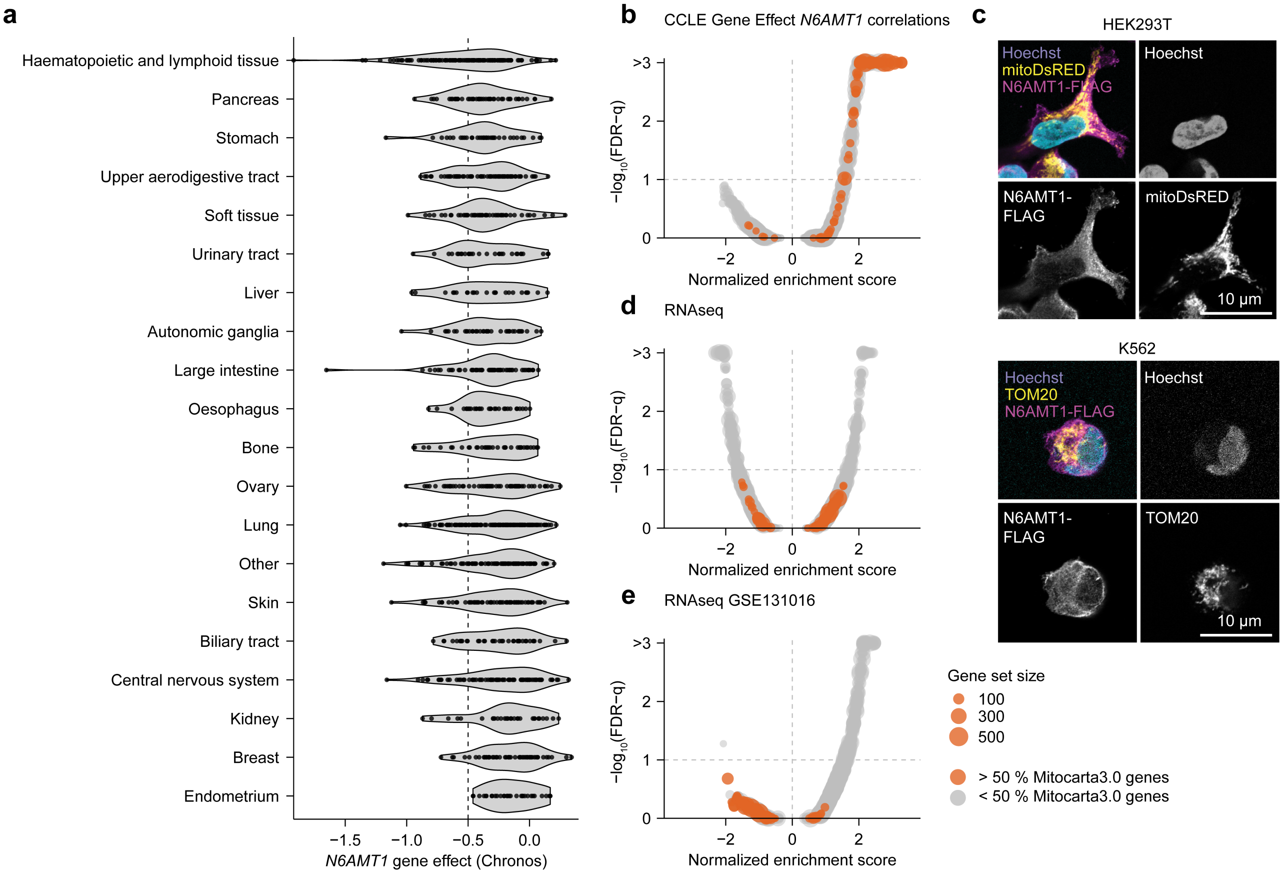

Figure S1. Additional analyses, related to Figure 1.

A) Violin plots showing distributions of *N6AMT1* CCLE Chronos scores by lineage. Lineages are ordered by increasing mean Chronos score.

B) Gene set enrichment analyses of correlations shown in Figure 1B. Gene sets with more than 50% MitoCarta3.0 genes are highlighted in orange.

C) Immunofluorescence analysis of N6AMT1 localization in HEK293T and K562 cells. Cells were transduced with N6AMT1-3xFLAG cDNA and immunolabelled with anti-FLAG. Mitochondria were visualized by mitoDsRED or anti-TOM20. Nuclei were visualized by Hoechst staining.

D-E) Gene set enrichment analyses of RNA-Seq shown in Figure 1F (C), and a publicly available RNA-Seq dataset comparing A549 control cell lines with siRNA-mediated *N6AMT1* knockdown (dataset GSE131016 in the Gene Expression Omnibus database) (D) showing no change in MitoCarta3.0 transcripts. Gene sets with more than 50% MitoCarta3.0 genes are highlighted in orange.

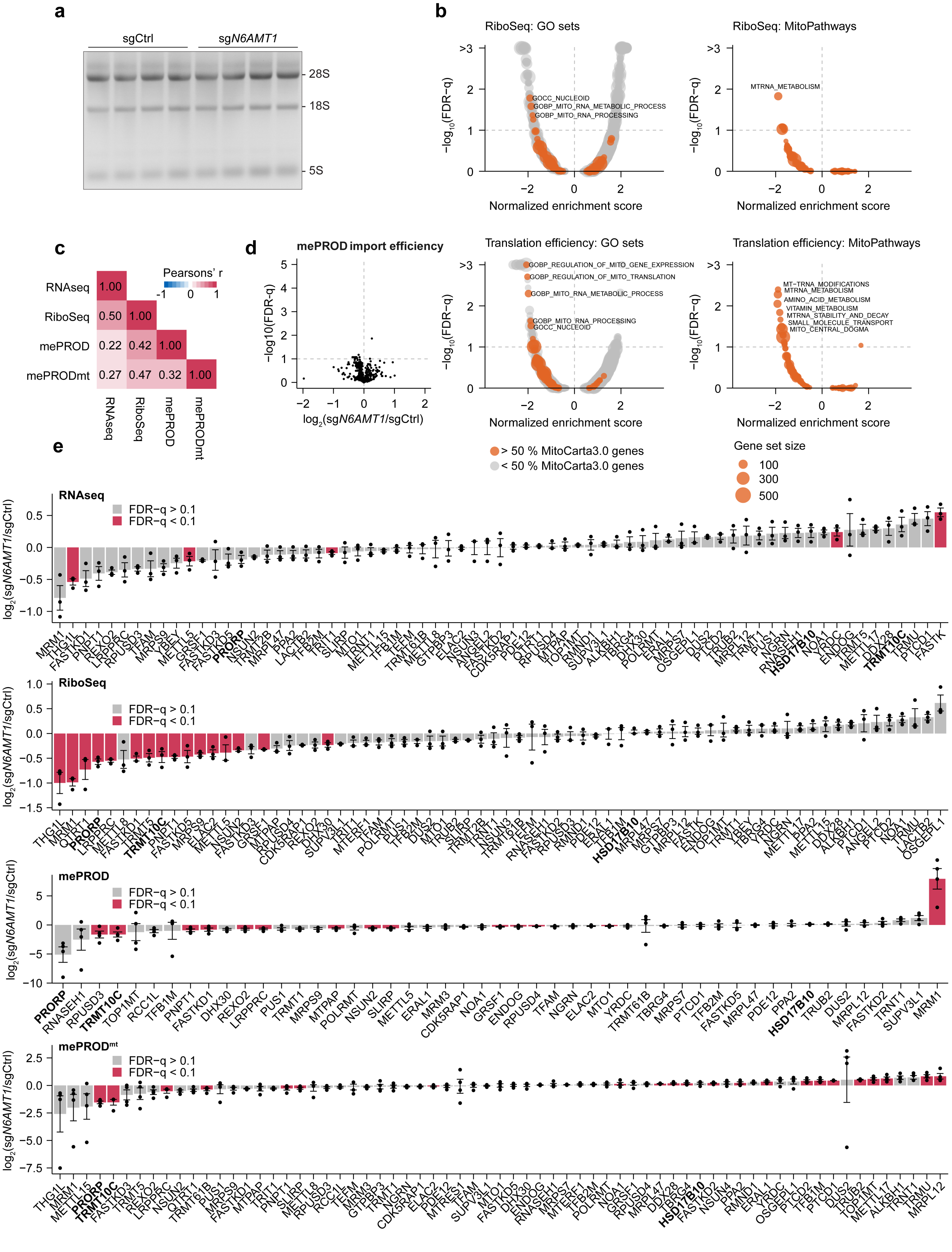
a
sgCtrl
sgN6AMT1
28S
18S
5S
b
RiboSeq: GO sets
RiboSeq: MitoPathways
GOCC_NUCLEOID
GOBP_MITO_RNA_METABOLIC_PROCESS
GOBP_MITO_RNA_PROCESSING
MTRNA_METABOLISM
−log10(FDR−q)
Normalized enrichment score
Translation efficiency: GO sets
Translation efficiency: MitoPathways
GOBP_REGULATION_OF_MITO_GENE_EXPRESSION
GOBP_REGULATION_OF_MITO_TRANSLATION
GOBP_MITO_RNA_METABOLIC_PROCESS
GOBP_MITO_RNA_PROCESSING
GOCC_NUCLEOID
MT−TRNA_MODIFICATIONS
MTRNA_METABOLISM
AMINO_ACID_METABOLISM
VITAMIN_METABOLISM
MTRNA_STABILITY_AND_DECAY
SMALL_MOLECULE_TRANSPORT
MITO_CENTRAL_DOGMA
> 50 % MitoCarta3.0 genes
< 50 % MitoCarta3.0 genes
Gene set size
100
300
500
c
Pearsons' r
RNAseq 1.00
RiboSeq 0.50 1.00
mePROD 0.22 0.42 1.00
mePRODmt 0.27 0.47 0.32 1.00
RNAseq RiboSeq mePROD mePRODmt
d
mePROD import efficiency
-log10(FDR-q)
log2(sgN6AMT1/sgCtrl)
e
RNAseq
RiboSeq
mePROD
mePRODmt
FDR−q > 0.1
FDR−q < 0.1
log2(sgN6AMT1/sgCtrl)

Figure S2. Additional analyses related to Figure 2.

A) RNA-agarose gel (basis for the quantification in 2A), comparing control to *N6AMT1*-depleted K562 cell lines 10 days post sgRNA transduction (n = 4 independent lentiviral infections per condition). Major ribosomal RNA (rRNA) peaks are indicated.

B) Gene set enrichment analyses of $\log_2$ (fold-change) values from ribosome profiling analysis (upper panels) and calculated translation efficiencies (ribosome-protected fragments / mRNA abundance; lower panels) based on Gene Ontology (GO) terms (left panels) or MitoPathways gene sets (right panels). Gene sets with > 50% MitoCarta3.0 genes are highlighted in orange.

C) Pearson's correlation coefficients between RNA-Seq, Ribo-Seq, mePROD, and mePROD^{mt} datasets.

D) Mitochondrial import analysis ($\log_2$-transformed fold-changes in mePROD^{mt} expression / mePROD expression ratios) in *N6AMT1*-depleted K562 as compared to control cells. n = 4 independent lentiviral transduction per condition.

E) Bar plots showing ranked $\log_2$ (fold-changes) of all genes belonging to the "mt-RNA metabolism" MitoPathways gene set in each of the four indicated datasets. Points represent log2 (fold-change) values for each independent replicate in the *N6AMT1*-depleted cells as compared to K562 cells. Error bars represent mean +/- standard error of the mean (n = 3-4 independent transduction per condition). Genes highlighted in red are significant at a false-discovery rate (FDR) of 0.1 (Benjamini-Hochberg).

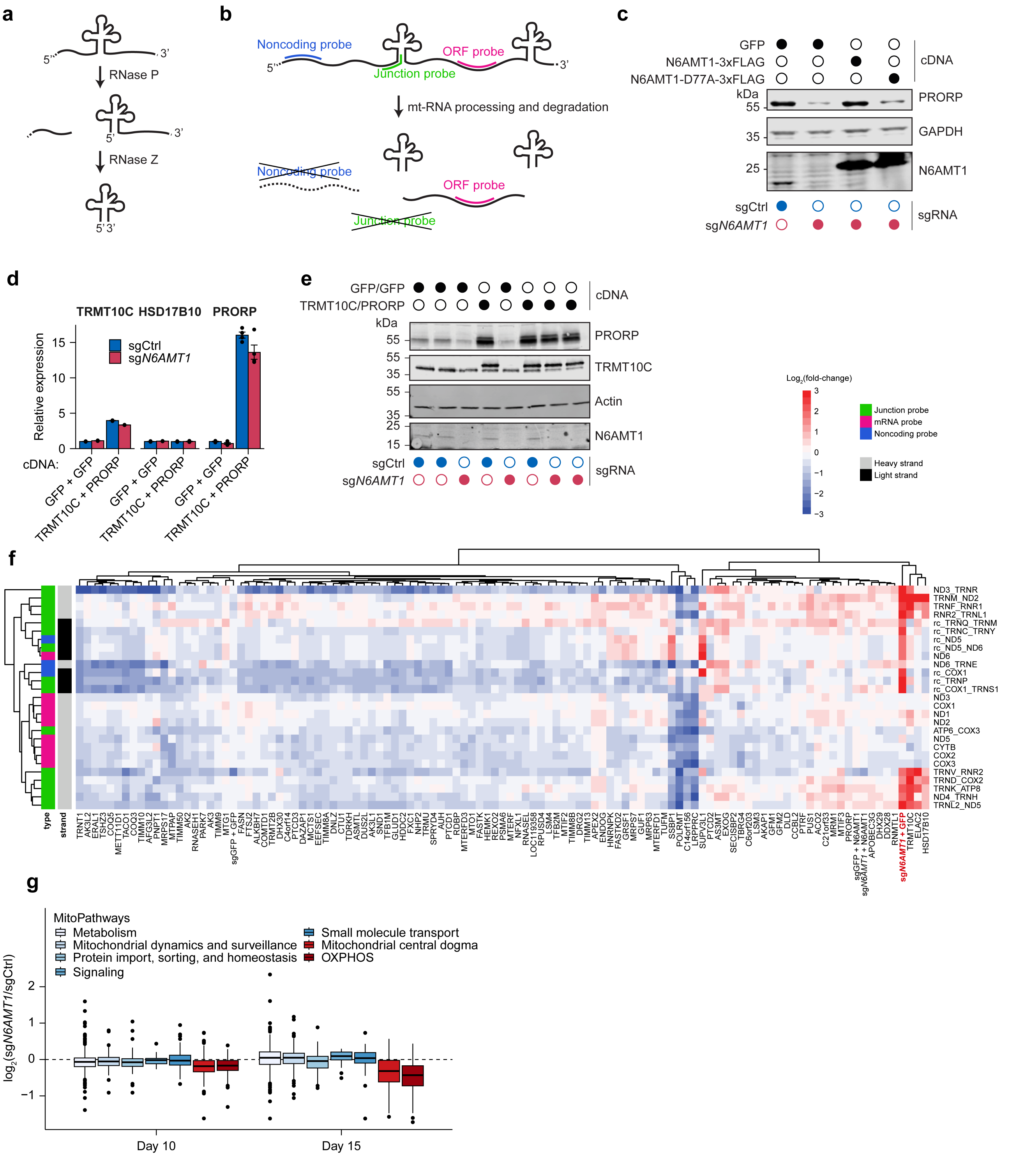
a
5'
3'
RNase P
5'
3'
RNase Z
5' 3'
b
Noncoding probe
ORF probe
Junction probe
5'
3'
mt-RNA processing and degradation
Noncoding probe
ORF probe
Junction probe
c
GFP
N6AMT1-3xFLAG
N6AMT1-D77A-3xFLAG
cDNA
kDa
55
35
25
PRORP
GAPDH
N6AMT1
sgCtrl
sgN6AMT1
sgRNA
d
TRMT10C
HSD17B10
PRORP
sgCtrl
sgN6AMT1
Relative expression
15
10
5
0
cDNA:
GFP + GFP
TRMT10C + PRORP
e
GFP/GFP
TRMT10C/PRORP
cDNA
kDa
55
35
25
15
PRORP
TRMT10C
Actin
N6AMT1
sgCtrl
sgN6AMT1
sgRNA
Log2(fold-change)
3
2
1
0
−1
−2
−3
Junction probe
mRNA probe
Noncoding probe
Heavy strand
Light strand
f
type
strand
ND3_TRNR
TRNM_ND2
TRNF_RNR1
RNR2_TRNL1
rc_TRNQ_TRNM
rc_TRNC_TRNY
rc_ND5
rc_ND5_ND6
ND6
ND6_TRNE
rc_COX1
rc_TRNP
rc_COX1_TRNS1
ND3
COX1
ND1
ND2
ATP6_COX3
ND5
CYTB
COX2
COX3
TRNV_RNR2
TRND_COX2
TRNK_ATP8
ND4_TRNH
TRNL2_ND5
sgN6AMT1 + GFP
TRMT10C
ELAC2
HSD17B10
g
MitoPathways
Metabolism
Mitochondrial dynamics and surveillance
Protein import, sorting, and homeostasis
Signaling
Small molecule transport
Mitochondrial central dogma
OXPHOS
log2(sgN6AMT1/sgCtrl)
2
1
0
−1
Day 10
Day 15

Figure S3. Additional analyses related to Figure 3.

A) Schematic representation of mitochondrial RNA processing by mitochondrial RNase P and Z. Mitochondrial RNase P is composed of TRMT10C (MRPP1), HSD17B10 (MRPP2), PRORP (MRPP3).

B) Illustration of MitoString to monitor mt-RNA processing defects. Three classes of probes targeting tRNA-mRNA junctions, noncoding regions, or regions within mRNAs are employed. Upon processing and degradation, junction-targeted probes or probes targeting non-coding regions are unable to bind their corresponding mt-RNA, thus showing reduced signal relative to mRNA-targeted probes.

C) Protein immunoblot showing mitochondrial RNase P subunit PRORP protein levels in control (sgCtrl) and *N6AMT1*-depleted (sg*N6AMT1*) K562 cells with introduction of cDNA encoding either GFP, wild type N6AMT1 or catalytically inactive N6AMT1-D77A.

D) qPCR analysis of RNase P-encoding transcripts in control or N6AMT1-depleted K562 cells, with introduction of either 2 different *GFP* cDNAs or *TRMT10C* + *PRORP* cDNA (n = 3 independent lentiviral infections).

E) Immunoblot showing mitochondrial RNase P subunits in control or N6AMT1-depleted K562 with the introduction of either 2 different GFP cDNAs or a combination of *TRMT10C* and *PRORP* cDNA.

F) Unsupervised clustering of MitoString data presented in Figure 3A with that of Wolf and Mootha (2014), illustrating the clustering of *N6AMT1*-depletion (sg*N6AMT1* + GFP) with depletion of mitochondrial RNase P (TRMT10C, HSD17B10) and RNase Z (ELAC2) proteins.

G) Proteomics protein abundance of mitochondrial proteins annotated as each of the six top-level pathway annotations in the MitoPathways database.

H) Proteomics protein abundance of mitochondrial OXPHOS proteins annotated according to OXPHOS complex as defined in the MitoPathways database.

I) Proteomics protein abundance of mitochondrial complex 5 subunits annotated according to complex V subdomains.

Uncropped immunoblots for Figure 1e

ID: MF071-5_20240214

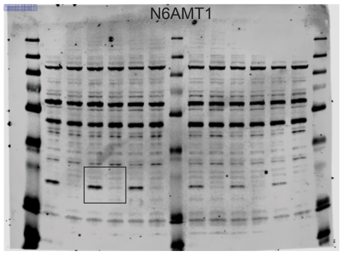

Primary antibody:
LS-C346278 (LSBio) 1:1,000
Secondary antibody:
IRDye® 800CW Goat anti-Rabbit
IgG Secondary Antibody 1:10,000

ID: MF071-5_20240222

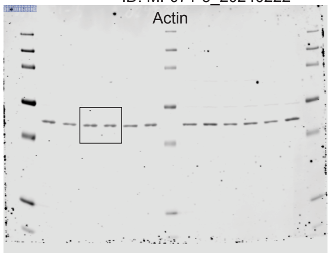

Primary antibody:
A1978 (Sigma) 1:1,000
Secondary antibody:
IRDye® 680RD Goat anti-Mouse IgG
Secondary Antibody 1:10,000

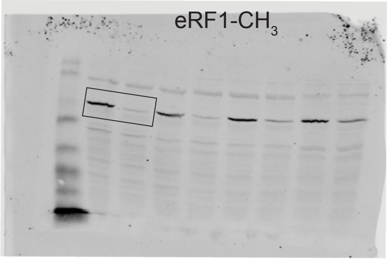

Primary antibody:
anti-eRF1-CH3

Secondary antibody:
IRDye® 800CW Goat anti-Rabbit
IgG Secondary Antibody 1:10,000

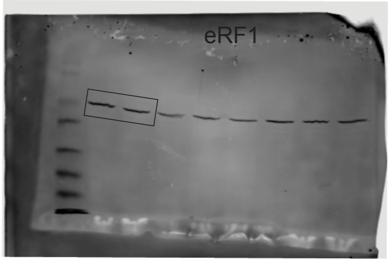

Primary antibody:
ab31799 (Abcam) 1:1,000

Secondary antibody:
IRDye® 680RD Goat anti-Mouse IgG
Secondary Antibody 1:10,000

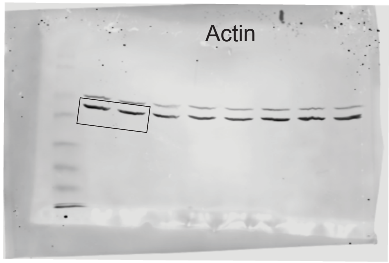

Primary antibody:
A1978 (Sigma) 1:1,000

Secondary antibody:
IRDye® 680RD Goat anti-Mouse IgG
Secondary Antibody 1:10,000

Uncropped immunoblots for Figure 1g

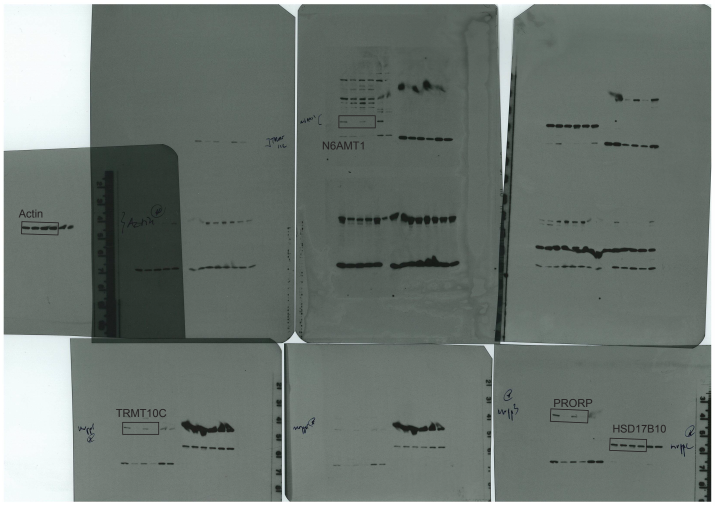

Uncropped immunoblots for Figure 3e

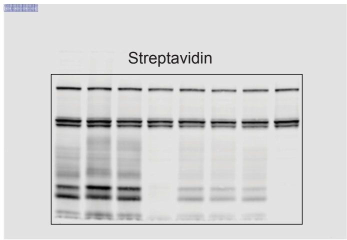

Detection by:
IRDye® 800CW Streptavidin1:10,000

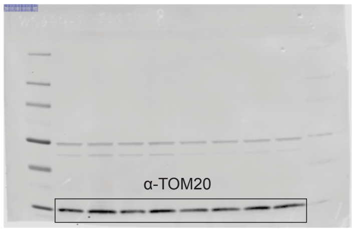

Primary Antibody:
42406 (Cell Signaling Technology), 1:1,000
Secondary antibody:
IRDye® 680RD Goat anti-Mouse IgG
Secondary Antibody 1:10,000

Figure S4. Uncropped immunoblots for Figures 1E, 1G and 3E.

Uncropped immunoblots for Figure S3c

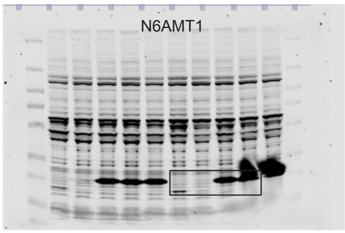

Primary antibody:
LS-C346278 (LSBio) 1:1,000
Secondary antibody:
IRDye® 800CW Goat anti-Rabbit
IgG Secondary Antibody 1:10,000

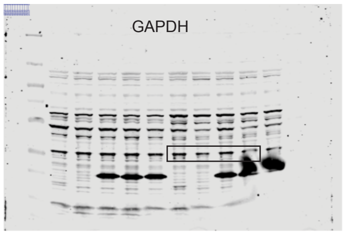

Primary antibody:
5174T (Cell Signaling Technology) 1:1,000
Secondary antibody:
IRDye® 800CW Goat anti-Rabbit
IgG Secondary Antibody 1:10,000

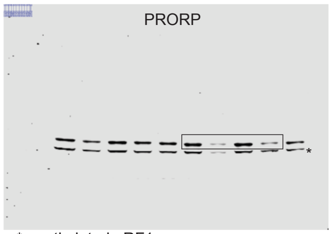

*: methylated eRF1

Primary antibody:
ab185942 (Abcam) 1:1,000
Secondary antibody:
IRDye® 800CW Goat anti-Rabbit
IgG Secondary Antibody 1:10,000

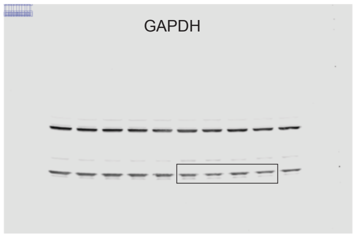

Primary antibody:
5174T (Cell Signaling Technology) 1:1,000
Secondary antibody:
IRDye® 800CW Goat anti-Rabbit
IgG Secondary Antibody 1:10,000

Uncropped immunoblots for Figure S3e

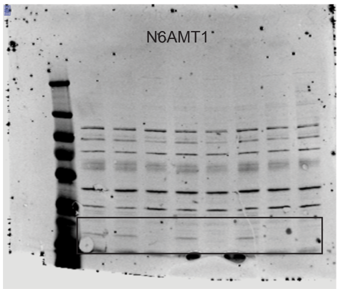

Primary antibody:
LS-C346278 (LSBio) 1:1,000
Secondary antibody:
IRDye® 800CW Goat anti-Rabbit
IgG Secondary Antibody 1:10,000

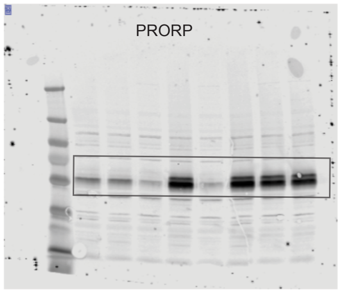

Primary antibody:
ab185942 (Abcam) 1:1,000
Secondary antibody:
IRDye® 800CW Goat anti-Rabbit
IgG Secondary Antibody 1:10,000

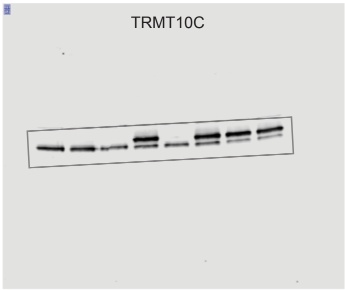

Primary antibody:
HPA036671 (Sigma) 1:1,000
Secondary antibody:
IRDye® 800CW Goat anti-Rabbit
IgG Secondary Antibody 1:10,000

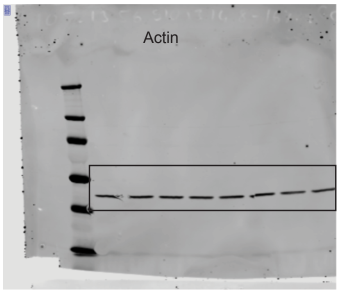

Primary antibody:
A1978 (Sigma) 1:1,000
Secondary antibody:
IRDye® 680RD Goat anti-Mouse IgG
Secondary Antibody 1:10,000

Figure S5. Uncropped immunoblots for Figures S3C and S3E.

Table S1. sgRNAs, cDNAs and primers used in this study

ID	Sequence	Type	Purpose
N6AMT1 sg1	CAGCGACGTGTACGAGCCCG	sgRNA	N6AMT1 depletion
N6AMT1 sg2	GGGTACAAGCTGCTGCCTCA	sgRNA	N6AMT1 depletion
Control sg1 (OR11A1)	GTGATGCCAAAAATGCTGGA	sgRNA	CRISPR control
Control sg2 (OR2M4)	CCATAAGGGACAGTTGACTG	sgRNA	CRISPR control
EGFP sg1	GGGCGAGGAGCTGTTCACCG	sgRNA	CRISPR control
N6AMT1-3xFLAG	ATGGCAGGGGAGAACTTCGCTACGCCGTTCCACGGGCACGTGGGCCGCGGGGCTTTTAGTGATGTTTATGAACCTGCTGAGGACACGTTTCTGCTTTTGGAC<br>GCGCTGGAGGCAGCGGCTGCCGAACTGGCAGGAGTGGAAATATGCCTGGAAGTAGGGTCAGGGTCTGGTGTAGTATCTGCATTCCTAGCCTCTATGATAGGC<br>CCTCAAGCCTTATATATGTGTACTGATATCAACCCTGAAGCGGCTGCCTGCACGCTAGAGACAGCACGCTGTAACAAAGTTCACATTCAACCAGTTATTACA<br>GATTTGGTCAAAGGCTTGCTACCAAGATTGACCGAAAAAGTTGATCTTCTGGTGTTTAATCCCCCCTATGTAGTGACTCCACCTCAAGAGGTAGGAAGTCAC<br>GGAATAGAGGCAGCTTGGGCTGGTGGCAGAAATGGTCGGGAAGTCATGGACAGGTTTTTTCCCCTGGTTCCAGATCTCCTTTCACCAAGAGGATTATTCTAT<br>TTAGTTACCATTAAAGAAAACAACCCAGAAGAAATTTTGAAAATAATGAAGACAAAAGGTCTGCAAGGAACCACTGCACTTTCCAGACAAGCAGGCCAAGAA<br>ACTCTTTCAGTCCTCAAGTTCACCAAGTCTGATTATAAAGATCATGATGGCGATTATAAAGATCATGATATTGATTATAAAGATGATGATGATAAATAATAG<br>TGAATGGCAGGGGAGAACTTCGCTACGCCGTTCCACGGGCACGTGGGCCGCGGGGCTTTTAGTGATGTTTATGAACCTGCTGAGGACACGTTTCTGCTTTTG<br>GACGCGCTGGAGGCAGCGGCTGCCGAACTGGCAGGAGTGGAAATATGCCTGGAAGTAGGGTCAGGGTCTGGTGTAGTATCTGCATTCCTAGCCTCTATGATA<br>GGCCCTCAAGCCTTATATATGTGTACTGATATCAACCCTGAAGCGGCTGCCTGCACGCTAGAGACAGCACGCTGTAACAAAGTTCACATTCAACCAGTTATT<br>ACAGATTTGGTCAAAGGCTTGCTACCAAGATTGACCGAAAAAGTTGATCTTCTGGTGTTTAATCCCCCCTATGTAGTGACTCCACCTCAAGAGGTAGGAAGT<br>CACGGAATAGAGGCAGCTTGGGCTGGTGGCAGAAATGGTCGGGAAGTCATGGACAGGTTTTTTCCCCTGGTTCCAGATCTCCTTTCACCAAGAGGATTATTC<br>TATTTAGTTACCATTAAAGAAAACAACCCAGAAGAAATTTTGAAAATAATGAAGACAAAAGGTCTGCAAGGAACCACTGCACTTTCCAGACAAGCAGGCCAA<br>GAAACTCTTTCAGTCCTCAAGTTCACCAAGTCTGATTATAAAGATCATGATGGCGATTATAAAGATCATGATATTGATTATAAAGATGATGATGATAAATAA<br>TAGTGA	cDNA	sgRNA-resistant
D77A_s	GCCATCAACCCTgaagcggctgcc	Primer	Introduce D77A mutation in N6AMT1 cDNA
D77A_as	AGTACACATATATAAGGCTTGAGGGCC	Primer	Introduce D77A mutation in N6AMT1 cDNA

Table S2. qPCR probes used in this study

ID	Source	Catalog number	forward primer	reverse primer	probe
TRNM-MT-ND2	Thermo Fisher Scientific	Custom probe	CCCATACCCCG AAAATGTTGGTTAT	CCTGCAAAGATGGTAGAGTAGATGA	CCCGTACTAATTAATCCC
MT-ND3-TRNR	Thermo Fisher Scientific	Custom probe	CCCTAAGTCTGG CCTATGAGTGA	AATGAGTCGAAATCATTCGTTTTGTTTAAACT	ACCAATTCGGTTCAGTCTAA
MT-RNR2-TRNL	Thermo Fisher Scientific	Custom probe	CCACACCCACC CAAGAACAG	AGTTTTATGCGATTACCGGGCT	CTGCCATCTTAACAAACC
TRNV-MT-RNR2	Thermo Fisher Scientific	Custom probe	CCACACCCACC CAAGAACAG	AGTTTTATGCGATTACCGGGCT	CTGCCATCTTAACAAACC
TRNM-MT-ND2	IDT	Custom probe	CCCATACCCCG AAAATGTTGGTTAT	CCTGCAAAGATGGTAGAGTAGATGA	CCCGTACTAATTAATCCC
MT-ND3-TRNR	IDT	Custom probe	CCCTAAGTCTG GCCTATGAGTGA	AATGAGTCGAAATCATTCGTTTTGTTTAAACT	ACCAATTCGGTTCAGTCTAA
MT-RNR2-TRNL	IDT	Custom probe	CCACACCCAC CCAAGAACAG	AGTTTTATGCGATTACCGGGCT	CTGCCATCTTAACAAACC
TRNV-MT-RNR2	IDT	Custom probe	CCACACCCACC CAAGAACAG	AGTTTTATGCGATTACCGGGCT	CTGCCATCTTAACAAACC
MT-RNR2	Thermo Fisher Scientific	Hs02596860_s1			
MT-ND2	Thermo Fisher Scientific	Hs02596874_g1			
MT-ND3	Thermo Fisher Scientific	Hs02596875_s1			
N6AMT1	Thermo Fisher Scientific	Hs01061650_g1			
PNPT1	Thermo Fisher Scientific	Hs01105971_m1			
IFNB1	Thermo Fisher Scientific	Hs01077958 s1			
USP18	Thermo Fisher Scientific	Hs00276441_m1			
IFIT1	Thermo Fisher Scientific	Hs03027069_s1			
GBP2	Thermo Fisher Scientific	Hs00894837_m1			
18S rRNA	Thermo Fisher Scientific	Hs03003631_g1			
TRMT10C	Thermo Fisher Scientific	Hs01933516_s1			
HSD17B10	IDT	Hs.PT.58.2288610	ACAGTTGACAGCTACATCCAC	CAAGCCAAGAAGTTAGGAAACAAC	/56-FAM/AGCCAGAGC/ZEN/TGTTT GCACATCCT/3IABkFQ/
PRORP	Thermo Fisher Scientific	Hs00206448_m1			

Table S3. Ribosome profiling adapters used in this study

ID	Sequence	Type	Purpose
oBZ207_profiling_bar11	CAAGCAGAAGACGGCATACGAGATgagcttGTGACTGGAGTTCAGACGTGTGCTCTTCCG	oligo	ribosome profiling PCR R
oBZ208_profiling_bar12	CAAGCAGAAGACGGCATACGAGATtttaagGTGACTGGAGTTCAGACGTGTGCTCTTCCG	oligo	ribosome profiling PCR R
oBZ209_profiling_bar13	CAAGCAGAAGACGGCATACGAGATaacttgGTGACTGGAGTTCAGACGTGTGCTCTTCCG	oligo	ribosome profiling PCR R
oBZ210_profiling_bar14	CAAGCAGAAGACGGCATACGAGATctggacGTGACTGGAGTTCAGACGTGTGCTCTTCCG	oligo	ribosome profiling PCR R
oBZ211_profiling_bar15	CAAGCAGAAGACGGCATACGAGATggtccaGTGACTGGAGTTCAGACGTGTGCTCTTCCG	oligo	ribosome profiling PCR R
oBZ212_profiling_bar16	CAAGCAGAAGACGGCATACGAGATtcaagtGTGACTGGAGTTCAGACGTGTGCTCTTCCG	oligo	ribosome profiling PCR R
oBZ287_profiling_F_NINI2	AATGATACGGCGACCACCGAGATCTACAC	oligo	ribosome profiling PCR F
oBZ407_cloning_linker_2_preA_rand	AppNNNNNNCACTCGGGCACCAAGGAC/3ddC/	oligo	preadenylated adaptor for ribosome profiling
oBZ408_RN3_linker2_RT	/5Phos/RNNNAGATCGGAAGAGCGTCGTGTAGGGAAAGAGTGTAGATCTCGGTGGTCGC/iSP18/TTCAGACGTGTGCTCTTCCGATCTGTCCTTGGTGCCCGAGTG	oligo	circularizable RT primer for ribosome profiling

Table S4. RNA-Seq adapters used in this study

ID	Sequence
TruSeq Adapter, Index 5	ACAGTG
TruSeq Adapter, Index 6	GCCAAT
TruSeq Adapter, Index 7	CAGATC
TruSeq Adapter, Index 8	ACTTGA
TruSeq Adapter, Index 9	GATCAG
TruSeq Adapter, Index 10	TAGCTT

Dataset S1 (separate file). Pearson's correlation coefficients obtained correlating N6AMT1 CCLE gene effect with each of the 15677 non-essential genes in the CCLE database across 1,095 cell lines.

Dataset S2 (separate file). Gene set enrichment analysis of Pearson's correlation data (from Dataset S1).

Dataset S3 (separate file). Output from DEseq analysis of RNA-Seq data.

Dataset S4 (separate file). Gene set enrichment analysis of $\log_2$(fold changes) of RNA seq analysis (Dataset S3) using the gene ontology database.

Dataset S5 (separate file). Gene set enrichment analysis of $\log_2$ (fold changes) of RNA seq analysis of GEO dataset GSE131016 using the gene ontology database.

Dataset S6 (separate file). Output from DEseq analysis of Ribo-Seq data.

Dataset S7 (separate file). Gene set enrichment analysis of $\log_2$(fold changes) of ribosome profiling dataset (Dataset S6) using the gene ontology database.

Dataset S8 (separate file). Gene set enrichment analysis of $\log_2$(fold changes) of ribosome profiling dataset (Dataset S6) using the MitoCarta MitoPathways database.

Dataset S9 (separate file). Gene set enrichment analysis of $\log_2$ (fold changes) of translation efficiencies (calculated from the Ribo-Seq and RNA-seq datasets) using the gene ontology.

Dataset S10 (separate file). Gene set enrichment analysis of $\log_2$ (fold changes) of translation efficiencies (calculated from the Ribo-Seq and RNA-seq datasets) using the MitoCarta MitoPathways database.

Dataset S11 (separate file). mePROD analysis of *N6AMT1*-depleted vs control K562 cell lines.

Dataset S12 (separate file). $mePROD^{mt}$ analysis of isolated mitochondria from *N6AMT1*-depleted vs control K562 cell lines.

Dataset S13 (separate file). Proteomics analysis of *N6AMT1*-depleted vs control K562 cell lines.
